# The First Electrochemical Cycle: State‐of‐Charge Dependent Formation and Evolution of the Solid Electrolyte Interphase on Hard Carbon Anodes in Sodium‐Ion Batteries

**DOI:** 10.1002/smtd.70958

**Published:** 2026-08-14

**Authors:** David Schäfer, Sven Daboss, Sarah Lang, Christine Kranz, Marcus Rohnke

**Affiliations:** ^1^ Institute of Physical Chemistry and Center for Materials Research Justus Liebig University Giessen Giessen Germany; ^2^ Institute of Analytical and Bioanalytical Chemistry Ulm University Ulm Germany; ^3^ CELEST Green Energy Lab Ulm Ulm University Ulm Germany

**Keywords:** AFM, correlative imaging, formation, hard carbon, sodium‐ion battery, solid electrolyte interphase, ToF‐SIMS

## Abstract

Understanding the solid electrolyte interphase (SEI) is central to improving the performance and longevity of sodium‐ion batteries. Here, we combine scanning electron microscopy (SEM), conductive atomic force microscopy (c‐AFM), energy‐dispersive x‐ray spectroscopy (EDS), time‐of‐flight secondary ion mass spectrometry (ToF‐SIMS), and scanning electrochemical microscopy (SECM) to investigate SEI formation on Hard Carbon (HC) composite electrodes. Unlike most literature, which analyses the SEI after multiple cycles, we examine different states of charge during the first galvanostatic cycles in sodium half‐cells using EC:PC with FEC additive and NaPF_6_ as the electrolyte. SEM reveals early surface film formation becoming continuous at full sodiation, while c‐AFM shows surface conductivity is strongly suppressed below 0.2 V, with only isolated conductive domains recovering after desodiation. EDS confirms accumulation of Na and O species on HC particles alongside persistent F components, identified as NaF by XPS. Depth‐resolved ToF‐SIMS reveals an SEI evolving from an oxide/hydroxide‐rich outer layer to a NaF‐dominated interphase, with Na_2_F^+^ signals increasing steadily through desodiation. SECM provides a functional link to these changes, as the apparent rate constant *κ* decreases sharply during sodiation and only partly recovers upon desodiation. These findings offer an in‐depth understanding of initial SEI dynamics in HC composite electrodes for sodium‐ion batteries.

## Graphical abstract

The formation and evolution of the solid electrolyte interphase on hard carbon electrodes is tracked at defined states of charge during the first galvanostatic cycle in sodium half‐cells. Combining SEM/EDS, XPS, ToF‐SIMS, SECM, and c‐AFM reveals how the SEI's morphology, chemistry, and interfacial reactivity develop during sodiation and partially reverse upon desodiation.

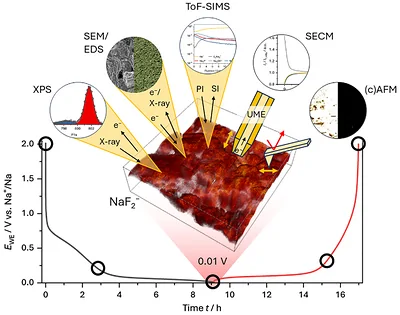

## 1 Introduction

Sodium‐ion batteries (SIBs) pose a valuable opportunity to fill the day‐to‐night gap and buffer grid overproductions concerning the utilization of renewable energy from, for example, solar and wind energy harvesting. Considering the high abundance and low cost of raw materials SIB prices may drop below the costs of lithium‐ion batteries (SIBs today: $125/kWh vs. LIBs today: $50–70/kWh) in the near future [1, 2]. SIBs are already playing an increasing role in covering world‐wide growing energy demands, but must not be understood as a competitor to state‐of‐the‐art lithium‐ion batteries (LIBs), which fulfil most of today's everyday mobile energy storage demands due to unmatched specific energies (LIBs ∼300 Wh/kg, SIBs ∼200 Wh/kg) [3, 4]. SIBs will rather fill the gap for applications with minor energy and power density needs but with high energy volume demands, for example, in grid storage applications [1].

Although SIBs are the subject of intensive research, there is still no complete agreement and understanding presented in the literature on factors determining the performance of SIBs. Firstly, the effect of the widely used hard carbon (HC) anode active material's structural properties and on sodium(‐ion) storage mechanisms concerning adsorption, intercalation, and pore filling is being discussed [5, 6]. Secondly, there are disagreements about the formation and stability of the lifetime‐limiting solid electrolyte interphase (SEI) in SIBs [7]. While earlier studies mainly focused on optimizing electrode materials, more recent efforts were directed towards tuning the electrolyte composition with the target of a defined stable SEI chemistry and functionality [8, 9].

The SEI is related to the reduction of the organic and inorganic components of the electrolyte during the first charge and discharge cycle(s). Commonly used electrolytes are cyclic esters like ethylene carbonate (EC), propylene carbonate (PC), linear esters like dimethyl carbonate (DMC) and diethyl carbonate (DEC) as well as ethers like diethylene glycol dimethyl ether (diglyme) or triethylene glycol dimethyl ether (triglyme). Additives such as fluoroethylene carbonate (FEC) increase the cyclability of sodium‐ion half‐cells for carbonate‐based electrolytes and are typically being reduced already at higher potentials versus Na^+^/Na than EC or PC to support the formation of NaF‐rich interphases. Additionally, FEC secures facile sodium deposition and extraction at the counter electrode by forming a passivation layer on the Na metal in sodium‐ion half‐cells. The latter also inhibits unfavorable reaction with PC at the sodium counter electrode, which organic solvents usually undergo, and thereby maintains functionality of the cell even at prolonged cycling [10].

Decomposition of the most common conductive salt NaPF_6_ likewise yields inorganic fluorides and Na*_x_* PF*_y_* O*_z_* species [11]. The exact sequence of reactions and their products depend on local potential, surface chemistry, and solvation‐conditions that make parallel reaction pathways difficult to separate and unambiguous description difficult [12, 13]. Compared with the more organic compounds based SEI in LIBs, the SEI in SIBs on HC composite electrodes tends to be more inorganic. That compositional shift means LIB‐derived additive rules cannot be fully transferred to SIBs. FEC can markedly improve SEI quality in small concentrations (e.g., 1.5%–3%) yet reduce it in higher concentrations (e.g., 10%), altering impedance and cyclability in opposite directions [13, 14, 15].

Various analytical techniques have been employed to study SEIs, each with specific advantages and limitations. x‐ray photoelectron spectroscopy (XPS) offers surface‐sensitive chemical state information but is limited in lateral resolution and sensitivity. Transmission electron microscopy (TEM) provides (sub)nanometer‐scale structural and morphological insights but may suffer from beam damage and only probes a tiny fraction of an electrode sample, which can be disadvantageous for inhomogeneous samples. Raman spectroscopy enables the identification of carbon and inorganic phases but lacks comprehensive chemical specificity for complex mixtures. The combination of complementary methods is therefore essential for building a more comprehensive insight into SEI formation [16]. Most works in the literature characterize the SEI after full cycling in the desodiated state, losing information on the SEI formation process and evolution within the very first cycle. This is partly explained by the SEI's high reactivity with atmospheric moisture, posing complications on sample handling. The postmortem bias complicates mechanism assignment and risks handling artifacts [9, 17]. Furthermore, discussions about SEI formation tend to be biased about the homogeneity across the electrode surface. Usually, single particles (TEM) of the electrodes or even smaller scales are subject to the evaluation of the SEI formation, while no attention is paid to spatial distribution of SEI components [18]. Therefore, we specifically address processes of the SEI formation in a laterally resolved and state‐of‐charge dependent manner at intermediate sodiation stages during the first cycle.

Scanning electron microscopy (SEM), conductive atomic force microscopy (c‐AFM), energy dispersive x‐ray spectroscopy (EDS), time‐of‐flight secondary ion mass spectrometry (ToF‐SIMS) and scanning electrochemical microscopy (SECM) were applied to follow HC surface evolution during galvanostatic charging and discharging (GCD) and connect chemical changes at the surface of the electrode to changes in physical properties of the interface [19, 20]. By combining these techniques with complementary spatial resolution and information depth, it is possible to track morphological, chemical, and functional changes with respect to conductivity at the HC composite electrode interface to form a holistic view of the initial SEI formation.

## 2 Results

The first electrochemical cycle is decisive for the long‐term performance of HC anodes, as the SEI is initiated and largely established within this single cycle [21]. Its composition, thickness, and lateral homogeneity, imprinted during this initial formation step, govern Coulombic efficiency, interfacial impedance, and cycling stability in all subsequent cycles [22]. Since SEI formation proceeds through transient, potential‐dependent reaction steps, these processes cannot be resolved by comparing only the pristine and fully cycled states. Intermediate stages along the first cycle must be probed directly [23, 24]. To this end, a combination of complementary analytical methods is applied here to capture how the HC surface and its SEI evolve during the first electrochemical cycle.

### 2.1 Electrochemical Considerations

First, we evaluated the electrochemical behavior of the HC composite electrodes, composed of 93 wt.% HC, 1.4 wt.% carbon black (C65), 1.87 wt.% carboxymethyl cellulose (CMC) and 3.73 wt.% styrene‐butadiene rubber (SBR). The galvanostatic cycling and corresponding differential capacity plots reveal distinct potential regions associated with the initial SEI formation [11, 14, 25] and the successive sodium insertion and extraction processes [26], which is consistent with previously published results. These characteristic features were used to define specific cut‐off potentials that represent the relevant stages of electrode evolution during the first cycle, at which HC samples were taken from the cells and subjected to postmortem analysis. Surface morphology, chemical composition and local conductivity are examined in detail. This approach enables a direct correlation between the electrochemical signatures observed in the GCD profiles and the physical and chemical changes occurring at the electrode‐electrolyte interface during the initial cycle.

**FIGURE 1:**
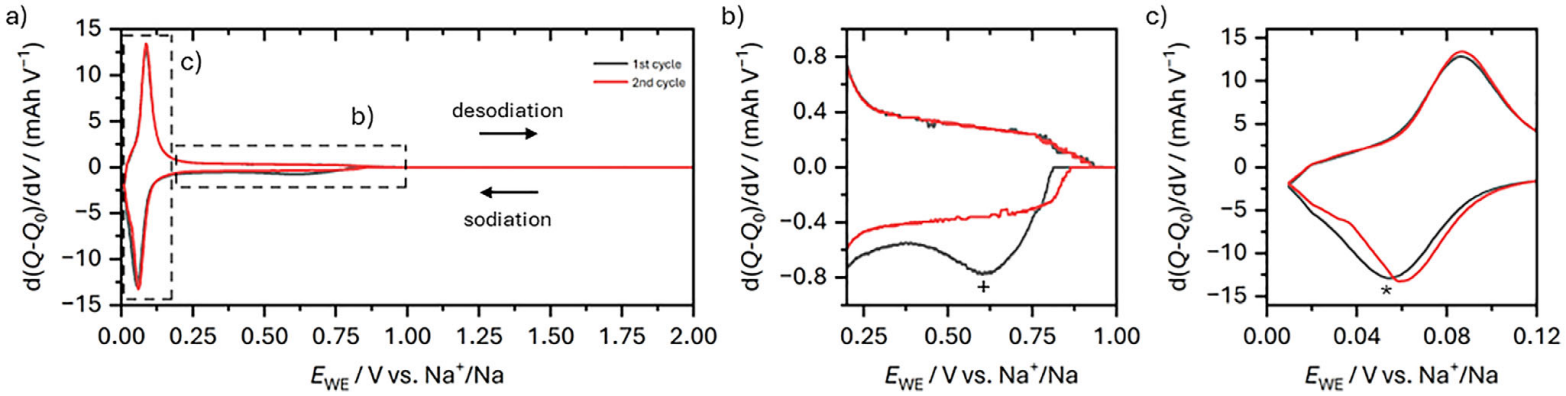
(a) d*Q*/d*V*‐plot calculated from GCD measurements of the first 2 cycles of HC electrodes cycled against sodium in a three electrode setup at 0.1 C (65 µA, 58 µA/cm^2^), (b) enlarged interval for the primary SEI forming potential at 0.6 V versus Na^+^/Na and (c) enlarged interval for the major sodium insertion potential at 0.05 V versus Na^+^/Na in the first cycle and 0.06 V versus Na^+^/Na from the second cycle.

Figure 1 shows the d*Q*/d*V* ratio versus the potential calculated from the first two cycles of a three‐electrode cell setup with sodium metal counter and reference electrode. This kind of plot offers information about the potentials at which reactions take place. Apparently, during the first sodiation process (negative values in Figure 1 of the black curve) virtually no current is measured until 0.8 V as a cause of different overpotentials that need to be overcome like desolvation of solvent molecules from sodium ions, charge transfer into the electrode and sodium extraction on the counter electrode. Below 0.8 V two peaks can be made out which are enlarged in Figure 1b,c.

The enlarged plots in Figure 1b,c reveal additional features for the first cycle at 0.6 V versus Na^+^/Na with a shoulder at ∼0.8 V versus Na^+^/Na, considered to be the initial irreversible SEI forming step, as no corresponding peak is detected during desodiation. This was shown to be the reduction peak of the FEC additive [11, 12]. Also, a shift between the first and second cycle towards higher potential versus Na^+^/Na during sodiation compared to the peak position of the first cycle at ∼0.06 V versus Na^+^/Na (* in Figure 1c) is attributed to a second irreversible process. The latter occurs due to additional overpotentials within the HC during the first cycle as the diffusion coefficient of Na^+^ in HC evolves as a function of state of charge (SOC) [27]. With no sodium stored in the HC in the pristine state, increased energetic efforts are necessary to initially insert sodium resulting in hysteresis effects between the first and following cycles [28]. This can be explained by a stretching of the *d* spacing of graphitic domains in the HC, where sodium is inserted and sodium cluster nucleation inside the pores, with both processes demanding initial energy contributions [27, 29].

**FIGURE 2:**
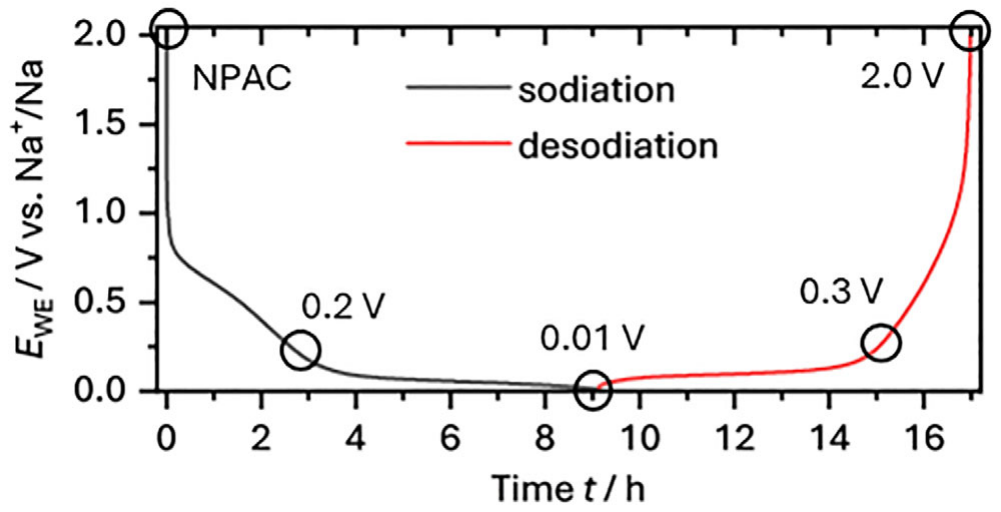
Potential versus time plot during GCD of HC composite electrodes cycled against sodium in a three electrode setup at 0.1 C (65 µA, 58 µA/cm^2^). The black line indicates sodiation and the red line desodiation. Black circles indicate the cut‐off potentials at which GCD was terminated, and the electrodes were subjected to surface investigation. The corresponding GCD curves for each cut‐off potential can be found in Figure S1.

The analyses in this work are focused on the sodiation stages corresponding to the aforementioned features shown in Figure 2, namely the non‐polarized assembled cell (NPAC), after the peak shown in Figure 1b (corresponding to the SOC after the sloping region in Figure 2 at 0.2 V vs. Na^+^/Na) to observe the FEC degradation, after the sodiation process (at 0.01 V vs. Na^+^/Na), after the oxidative desodiation plateau at 0.3 V versus Na^+^/Na and at full oxidation in the desodiated state (at 2.0 V vs. Na^+^/Na). In a first step, SEM and c‐AFM studies were performed to visualize changes at the interface, before EDS, ToF‐SIMS depth profiles and XPS measurements were conducted to investigate the chemical composition of the formed interphase.

### 2.2 Interface and Interphase Analyses

In the following, comparative image series are shown to present surface changes during the first polarization cycle. SEM and c‐AFM measurements on HC composite electrode are shown at several sodiation stages as previously marked in Figure 2. Also, interphase analyses by EDS elemental mappings and ToF‐SIMS depth profiles of the SEI layer are discussed.

**FIGURE 3:**
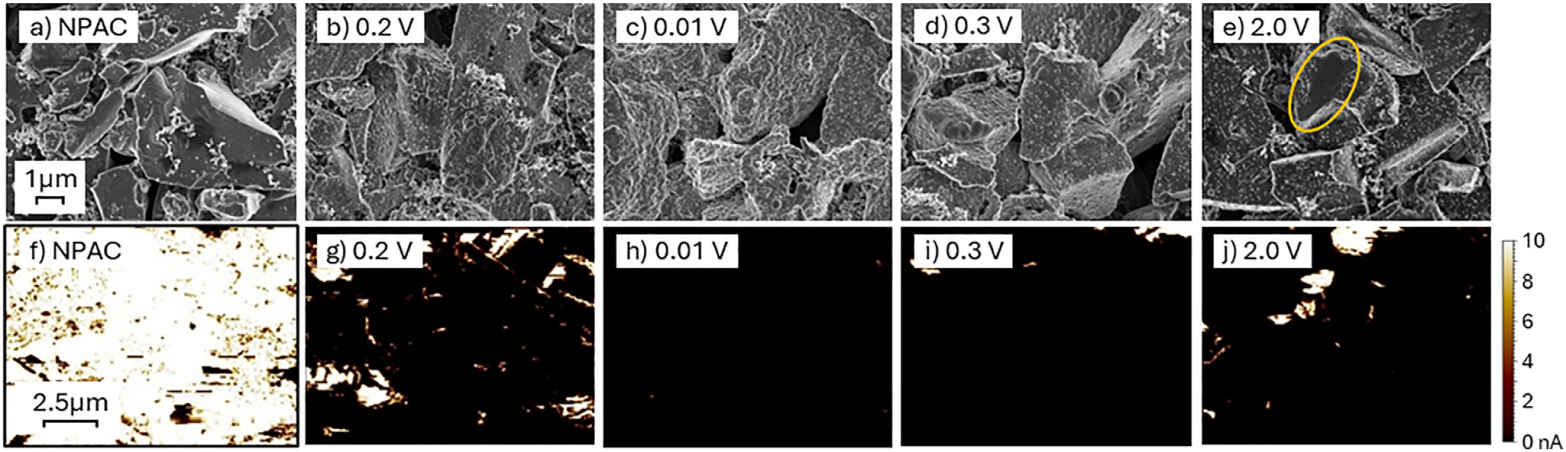
SEM images (a–e) and c‐AFM conductivity maps (f–j) of HC surfaces at different voltages of the same electrodes as in Figure 4. Increasing coverage of the particle surfaces in SEM images correlate closely with decreasing conductivity and vice versa as indicated by the circle in (e). Corresponding topography maps are displayed in Figure S4, confirming that the measured contrasts arise from actual conductivity variations rather than artifacts.

**FIGURE 4:**
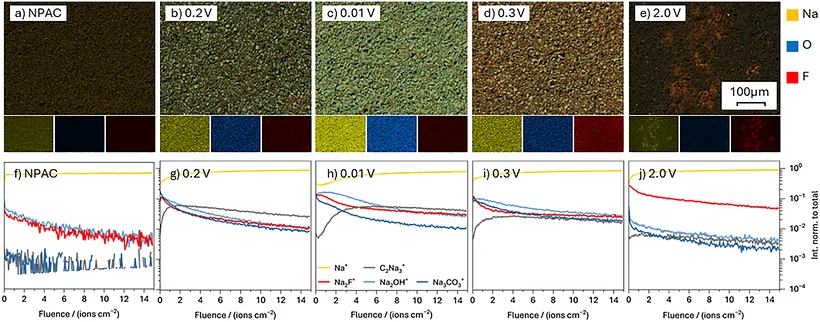
EDS elemental overlay maps (RGB) and corresponding single element maps (a–e) and ToF‐SIMS depth profiles (f–j) of HC electrodes at different voltages of the same electrodes as in Figure 3. Greenish coverages of the electrodes surfaces display simultaneous Na and O abundance, while during desodiation, these components disappear to give way to irregular NaF (orange) features. The depth profiles show a gradual increase of the Na_2_F^+^ concentration compared to O‐containing components. The latter developing a thicker layer at low potentials, which regresses during desodiation. C_2_Na_3_^+^ is assigned as a fragment of sodiated carbon and therefore marks the interface between SEI and HC particle.

SEM micrographs (Figure 3a–e) and c‐AFM conductivity maps (Figure 3f–j) together illustrate the morphological and conductivity evolution of the HC composite electrode surface at different electrochemical states, recorded on separate samples cut from the same electrode at the respective cut‐off potentials to ensure direct comparability. While the rough electrode morphology poses a challenge for conductive AFM and frequently induces tip artifacts, these localized features clearly correlate with the electrochemical state. The unpolarized electrode (NPAC, Figure 3a,f), a sample from a non‐polarized assembled cell, shows clean particle surfaces with only minor residuals from the electrode processing and a correspondingly homogeneous conductivity across the surface. Upon initial sodiation to 0.2 V versus Na^+^/Na (Figure 3b,g), the surfaces become visibly roughened on the nanoscale and covered with heterogeneous deposits, indicative of early electrolyte decomposition and nucleation of interphase species. In parallel, the conductivity vanishes almost completely, indicating the rapid onset of passivating surface reactions. At full sodiation (0.01 V vs. Na^+^/Na, Figure 3c,h), the HC particles are completely covered by a dense surface layer that smooths out the underlying particles and suggests the formation of a continuous SEI film, consistent with the essentially suppressed current response of an electronically insulating interphase. During desodiation at 0.3 V versus Na^+^/Na (Figure 3d,i), the SEI remains intact but appears more compact and conformal, with the apparent thickness decreased, while the current response stays suppressed across this deeper desodiation regime. After complete desodiation at 2.0 V versus Na^+^/Na (Figure 3e,j), the surfaces show irregular coverage, with some fractured regions pointing toward SEI instability and partial re‐exposure of the HC particle surfaces where the coverage appears chipped off (circled in Figure 3e); correspondingly, localized conductive spots reappear in the c‐AFM map on the same length scale as these scaled‐off regions, confirming partial SEI degradation and re‐exposure of the conductive HC surface. Figures S2,S3 in the supplementary information include SEM images and c‐AFM measurements from additional cut‐off potentials supporting these trends. Taken together, these observations demonstrate that the progressive formation, densification, and partial degradation of the SEI is highly potential‐dependent. The dynamic SEI evolution critically alters local electron transport pathways at the HC‐electrolyte interface, raising the question of which chemical species drive this potential‐dependent restructuring. To address this, the following EDS and ToF‐SIMS analyses resolve the elemental composition and lateral and in‐depth distribution of the SEI across the same electrochemical states.

EDS mapping (Figure 4a–e) provides complementary insight into the elemental composition and distribution of key elements (O, F, Na) across the HC electrode at different sodiation stages. Depending on the penetration depth of the high energetic electrons and the respective excitation bulb, the EDS data contains information about the first micrometres of the samples. Since the SEI is expected to be significantly thinner, the analytical information also includes volume fractions of HC. In the NPAC electrode (Figure 4a), only a faint oxygen signal is detected, originating from surface oxides or residual binder components, while fluorine and sodium are virtually absent, consistent with the unpolarized state of the assembled cell. Upon initial sodiation to 0.2 V versus Na^+^/Na (Figure 4b), oxygen‐rich domains become more uniformly distributed across the electrode, reflecting the early stages of electrolyte decomposition and SEI nucleation. At full sodiation (0.01 V vs. Na^+^/Na, Figure 4c), the O signal dominates, giving rise to a homogeneously greenish coloration, which suggests the formation of a continuous, oxygen‐rich SEI layer, most likely containing sodium oxides (Na_2_O) and carbonates (Na_2_CO_3_), in accordance with typical decomposition pathways of EC/PC‐based electrolytes [10, 30]. During desodiation at 0.3 V versus Na^+^/Na (Figure 4d), the fluorine signal (red) becomes more pronounced, indicating relative enrichment of fluorinated decomposition products. Notably, after complete desodiation at 2.0 V versus Na^+^/Na (e), sodium is still clearly detectable (yellow), highlighting residual Na‐containing species trapped within the HC. The co‐localization of Na with F in this state suggests a persistent inorganic‐rich SEI, in which NaF is the major constituent, which was previously observed on model electrodes [31]. This is substantiated by XPS measurements of the fluorine interval at the different sodiation stages (Figure S5). A dominating signal at 685 eV assigned to NaF [30] can clearly be identified for each sodiation stage, while only one second signal which was assigned to NaPF_6_ is detected in minor amounts for the NPAC sample and after one full cycle, potentially due to insufficient rinsing of the electrode. Overall, the EDS maps underline that SEI formation proceeds from an oxygen‐dominated initial stage during sodiation toward a more inorganic, Na‐F enriched layer upon desodiation, consistent with the conductivity suppression observed in c‐AFM and the morphological evolution seen in SEM.

ToF‐SIMS depth profiling was performed in addition to follow the chemical evolution of the SEI with cycling, enabling the identification of key fragments and their relative distribution as a function of sputter fluence on the nm scale. Identification of the SEI forming components was done by the evaluation of mass spectra as shown in Figure S6.

The surface of the sample NPAC (Figure 4f) is dominated by weaker signals originating from the Na‐containing binder (Na^+^,Na_2_OH^+^ from sodiated CMC binder) and minor residues of conductive salts (Na_2_F^+^). The fluence‐dependent decay is shallow, indicating only trace carbonate at the outermost surface prior to polarization. All Na‐containing fragments show sharply increased intensities at the sample surface in Figure 4g (0.2 V vs. Na^+^/Na) that decay with sputter fluence, evidencing interphase nucleation. The carbonate‐related signal (Na_3_CO_3_^+^) rises relative to NPAC and decays faster than F‐containing fragments, consistent with an oxygen‐rich, outer SEI formed by initial electrolyte reduction. In contrast, Na_2_F^+^ shows a pronounced relative enrichment at and beneath the topmost layers and decays more gradually, already hinting at an emerging inorganic F‐rich component (NaF) layer. At full sodiation, (0.01 V vs. Na^+^/Na, Figure 4h), the interphase becomes chemically layered. The carbonate‐derived fragment (Na_3_CO_3_^+^) peaks near the surface but drop quickly with depth, whereas Na_2_F^+^ increases further in relative intensity and maintains a broad, slowly decaying tail, indicating NaF accumulation and/or densification within the near‐surface region. Na^+^ also remains elevated, consistent with Na‐rich inorganic domains and trapped Na within the SEI as well as stored Na^+^ within the HC. This composition aligns with the c‐AFM observation of near‐complete electronic passivation at low potentials. Desodiation from full sodiation at 0.01 V versus Na^+^/Na to intermediate desodiation at 0.3 V versus Na^+^/Na and full desodiation at 2.0 V versus Na^+^/Na (Figure 4i,j) shows diminishing O‐containing contributions, while Na_2_F^+^ remains comparatively intense, pointing to relative enrichment of NaF as the more stable SEI constituent. Carbonate and hydroxide signatures are comparatively suppressed, indicating almost complete desolvation or disappearance of the respective SEI components. The persistent Na^+^ and NaF‐signals corroborate irreversible Na retention and a chemically robust, F‐rich interphase. Because the signals are normalized to the total ion signal at each measurement step, the systematic rise of relative Na_2_F^+^ intensity throughout sodiation and its sustained prominence through desodiation reflects a compositional shift toward an inorganic, NaF‐rich SEI. Carbonate‐ and hydroxide‐like species are most prominent during low‐potential growth but recede within the desodiation half‐cycle, whereas Na_2_F^+^ becomes the dominant component of the forming interphase. Together with SEM (morphology) and c‐AFM (loss of conductivity), the ToF‐SIMS profiles provide a consistent indication of the rapid initial formation of an O‐rich layer, followed by enrichment and long‐lived retention of an inorganic NaF‐rich inner SEI that governs interfacial transport on subsequent cycles.

It should be noted that ToF‐SIMS depth profiles, as shown here, are subject to matrix effects. Within the SEI regions of the investigated samples, the predominant presence of sodium oxides, carbonates, and fluorides was assumed to produce comparable matrix effects, making semi‐quantitative comparisons across samples reasonable. This assumption, however, does not hold upon entering the HC particle regime, where the differing chemical environment complicates such comparisons. For this reason, the analysis was limited to intensity comparisons within the SEI regions.

**FIGURE 5:**
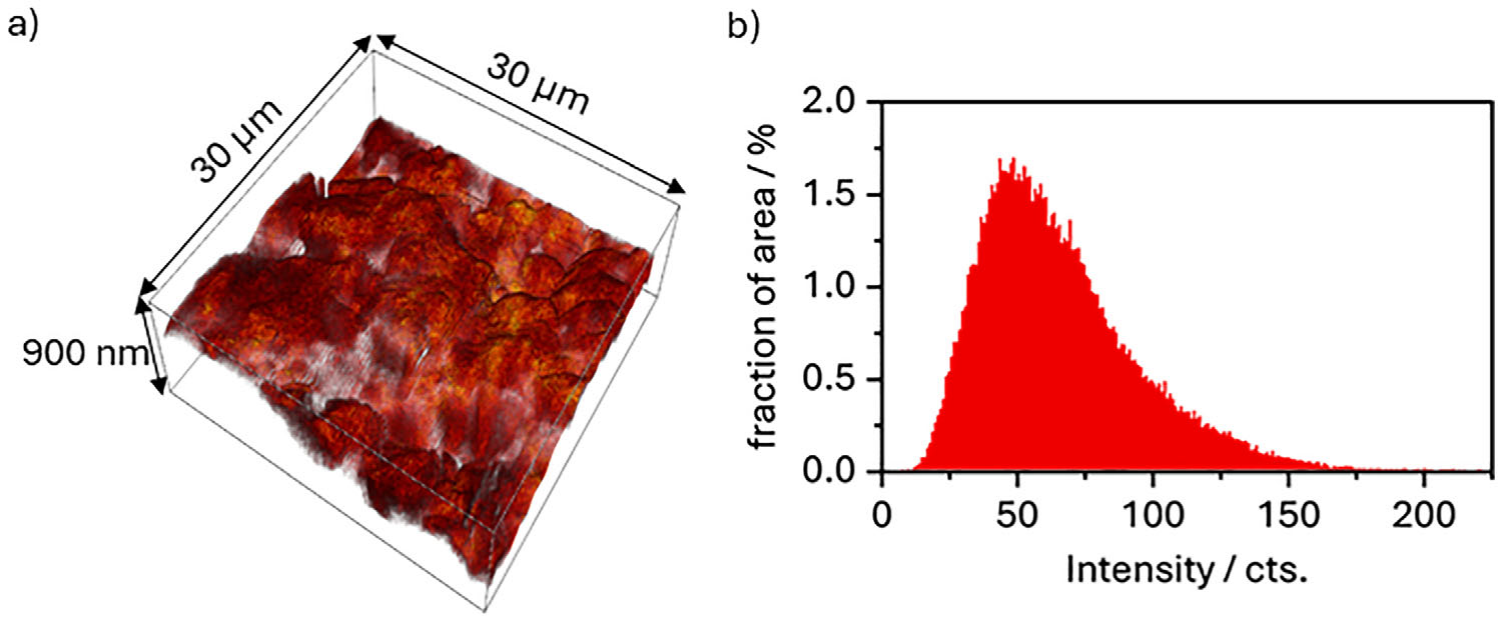
(a) Three‐dimensional rendering of the mass fragment Na_2_F^+^ from the topography‐corrected SIMS dataset acquired over a 30 × 30 µm^2^ area on an HC electrode sodiated to 0.01 V. Pronounced surface roughness and non‐uniform fragment distribution are evident across the analyzed surface. (b) Histogram of integrated Na_2_F^+^ fragment intensity per (x,y)‐column, illustrating a strongly skewed distribution indicative of substantial lateral heterogeneity in the NaF‐rich layer.

To further assess the lateral homogeneity of the presumptive NaF layer, ToF‐SIMS depth profiling was performed in combination with AFM‐derived topography correction. This correlative approach exploits the high lateral resolution of ToF‐SIMS while accounting for the pronounced surface roughness by applying laterally resolved depth correction based on AFM. The three‐dimensional reconstruction of the topography corrected dataset (Figure 5a) reveals a strongly heterogeneous surface morphology across the 30 × 30 µm^2^ analysis area, underscoring the necessity of topography correction for reliable depth‐resolved signal interpretation. Using Na_2_F^+^ as a representative mass fragment for the SEI, the coverage of the HC particles with SEI can be clearly detected.

The intensity histogram of the Na_2_F^+^ mass fragment (Figure 5b) exhibits a strongly right‐skewed distribution. Here, each pixel‐column refers to the depth‐integrated Na_2_F^+^ signal of a single lateral pixel, that is, the full stack of voxels along the sputter direction at that position in the 3D rendering. The skew therefore indicates that the majority of these columns contribute only weakly to the overall Na_2_F^+^ signal, while a smaller fraction reaches substantially higher integrated intensities, reflecting a laterally inhomogeneous distribution of NaF across the analyzed area. This behavior suggests the presence of a laterally heterogeneous NaF layer, in which regions of enhanced signal correspond either to locally increased layer thickness or to a higher volumetric NaF density, respectively. The continuous distribution of non‐zero values suggests SEI presence across the whole surface, while the broad intensity spread indicates substantial local variation.

### 2.3 Quantification

**FIGURE 6:**
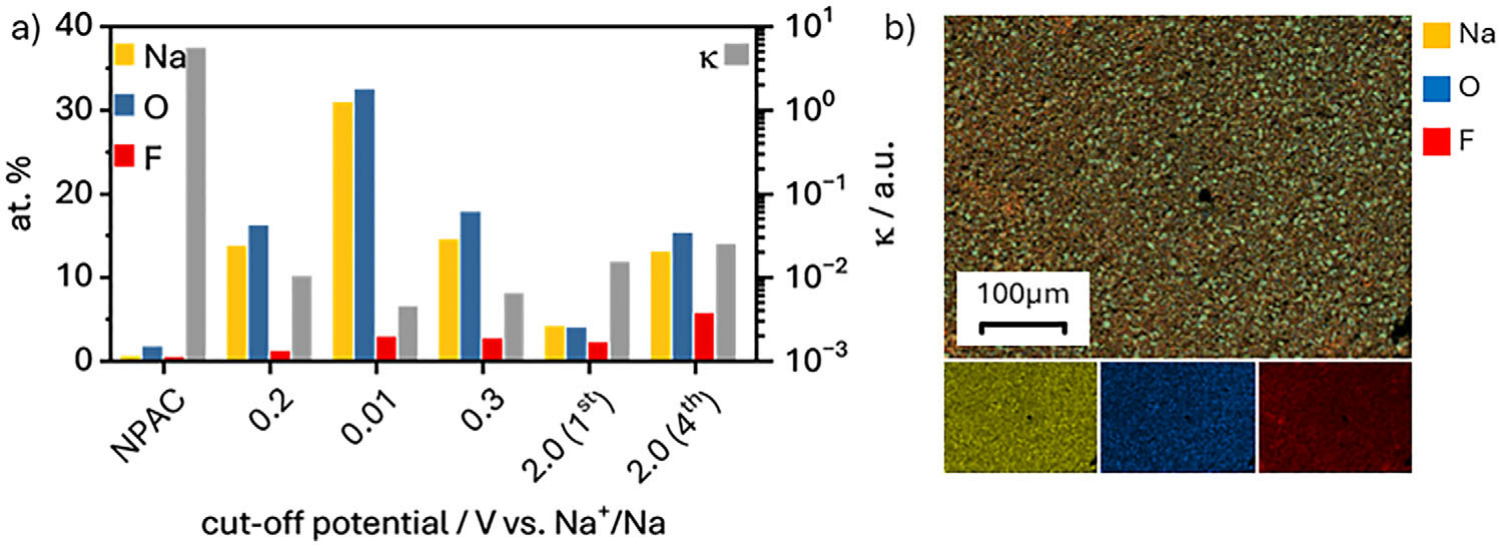
(A) Atomic percentages of Na, O, and F obtained from EDS (cf. Figure 4) plotted together with dimensionless heterogeneous rate constant *κ* from SECM (Figure S7). Na and O contents increase from 0.2 V versus Na^+^/Na during sodiation and decrease again during desodiation, whereas F remains constant once formed. The inverse correlation between *κ* and surface coverage indicates that the progressive formation of Na‐, O‐, and F‐containing SEI layers suppresses electron transfer, with persistent fluorine‐rich species maintaining interfacial insulation even after full desodiation. (b) EDS elemental mapping RGB overlay and single element maps of Na, O and F of the HC surface after 4 cycles. Greenish coverages of the electrode surface display simultaneous Na and O abundance, features of orange display NaF occurrence.

Figure 6 summarizes the quantitative EDS analysis of the electrode surface composition as a function of cut‐off potential, highlighting the evolution of oxygen, sodium, and fluorine contents. These values should be interpreted with care, since EDS quantification of light elements is associated with considerable uncertainties. However, semi‐quantitative trends can be unambiguously identified for the correlation with ToF‐SIMS depth profile intensities described above. The combined evaluation of EDS‐derived surface composition and SECM‐derived rate constant *κ* values (Figure 6, grey bars) establishes a clear relationship between chemical coverage and electronic conductivity of the HC electrode surfaces. The dimensionless heterogeneous rate constant *κ* describes the change in tip current as the SECM probe approaches the electrode surface and thus reflects the local electron‐transfer (electronic) conductivity. For a fully quantitative assessment, these values can be converted to effective rate constants (*κ*_eff_) using the mediator's diffusion coefficient and the tip radius (see Section S5 in the Supporting Information for a table of *κ*_eff_ values). However, to facilitate a direct, scale‐independent comparison across different samples and cycling states, the normalized dimensionless form *κ* is primarily discussed here. High *κ* values correspond to positive feedback, where the surface is electronically conductive and facilitates regeneration of the redox mediator, resulting in an increased tip current. In contrast, low *κ* values indicate negative feedback, characteristic of insulating or passivated surfaces, where electron transfer is hindered and the tip current decreases. Accordingly, variations in *κ* directly mirror the extent of surface passivation and SEI formation during cycling [32].

]In the pristine and NPAC states, *κ* > 1 reflects facile electron transfer across a largely uncoated carbon surface, in line with the low Na, O, and F concentrations measured by EDS. With progressive sodiation to 0.2–0.01 V versus Na^+^/Na, *κ* decreases by several orders of magnitude, inversely proportional to a steep rise in Na and O contents at the surface. This inverse correlation demonstrates that the build‐up of an Na‐ and O‐rich SEI directly suppresses electronic conductivity by masking the underlying carbon. Notably, F concentrations remain at a low but constant level across the entire cycle, suggesting the early formation of a persistent, fluorinated component, most likely NaF, which contributes disproportionately to electronic insulation. During desodiation (0.01–2.0 V vs. Na^+^/Na), Na and O contents measured by EDS gradually decline, indicating partial removal or restructuring of the SEI, while *κ* shows only a modest recovery. This mismatch reveals that although the chemical surface composition becomes depleted in Na and O quantities, the presence of stable F‐containing species and a compact inorganic matrix maintain high interfacial resistance. Summing up, the trends of two independent methods (EDS and SECM) confirm that SEI evolution is the primary factor governing the transition from a conductive to an insulating surface and highlight the role of fluorinated species in limiting conductivity recovery even at high potentials. This is substantiated by further EDS and SECM measurements after 4 cycles (Figure 6a “2.0 (fourth)”) which show an increased amount of degradation products by more intense Na, O, and F signals, but a virtually constant *κ* value compared to the first cycle (Figure 6a “2.0 (first)”). It can also be made out in the element maps in Figure 6b, that the distribution of the degradation products seems to be spread laterally significantly more homogeneous, especially the fluorine distribution compared to Figure 4e.

## 3 Discussion

While SEI formation in LIBs has been extensively studied, SIBs exhibit a markedly different interphase chemistry due to larger ion size, lower reduction potentials, and differing salt‐solvent stability. Yet despite this growing interest, most experimental SEI studies on HC anodes have characterized the interphase only after completed cycles, with some focusing on the first cycle as a reference point [8, 9, 10, 11, 12, 18, 19, 20, 29, 30] and others tracking evolution across multiple cycles [8, 9, 12, 18, 19, 20, 28, 29, 30]. Understanding not only SEI formation but also its temporal evolution during the first cycle is thus crucial to improve Coulombic efficiency and extend calendar life. The multi‐method characterization presented here provides a comprehensive picture of SEI formation on HC composite electrodes during the first electrochemical cycle in sodium‐ion battery half‐cells.

SEM imaging revealed the progressive coverage of carbon particles by decomposition products, having already set in majorly at 0.2 V versus Na^+^/Na, becoming continuous at 0.01 V versus Na^+^/Na, and exposing particle surface during desodiation. This adds imaging evidence about dense, inorganic‐rich SEI films on HC to reports in literature of rapid electrolyte reduction below 1.0 V vs Na^+^/Na [33]. Because most reports in the literature primarily focus on the post‐desodiation state, they mainly describe the surfaces of largely re‐exposed HC, as shown here in the SEM images after desodiation at 2.0 V versus Na^+^/Na. However, this captures only part of the picture. Intermediate stages indicate ongoing chemical changes that could allow to tune the stability of the SEI. This dynamic view is further supported by reports that a homogeneous SEI layer forms only over the course of several cycles, underlining that the interphase observed in the desodiated state does not represent a static endpoint [11].

SEI stability governs long‐term capacity retention and self‐discharge. Mogensen et al. [34] showed that the SEI in sodium half‐cells is more soluble than in lithium, causing rapid open‐circuit capacity loss via dissolution of interphase fragments, and that FEC suppresses this dissolution and reduces self‐discharge. The nature of the SEI is highly dynamic during the first cycle which we addressed with our studies and therefore could contribute to the understanding of the chemical evolution of the SEI layer during the first cycle.

EDS maps confirmed the accumulation of Na and O on the electrode during sodiation, with maxima at 0.01 V versus Na^+^/Na, while F appeared early and remained constant throughout cycling. The anti‐proportional trend between C and Na/O signals indicates progressive masking of the carbon surface. This agrees with prior studies that identified Na_2_CO_3_ species as dominant early SEI products [35, 36]. Furthermore, Ma et al. [37] measured SEI‐component solubility after cycling in 1 M NaPF_6_/PC, reporting a combined NaF/Na_2_CO_3_ solubility of 8.167 mg/L by ICP‐OES, while EC‐based electrolytes showed even lower values, and pre‐saturating with NaF or Na_2_CO_3_ had no effect on the initial capacity loss. Applied to the 70 µL electrolyte volume used in this study, this corresponds to at most ∼14 nmol of dissolved species in a worst‐case saturated solution. The persistence of F even after desodiation, thus, may suggest the formation of stable NaF domains to some extent, while other domains presumably are prone to delamination rather than dissolution. Such dynamics could underlie the low initial Coulombic efficiency and require stabilizers like FEC to suppress repetitive electrolyte decomposition. This also implies the need for more than one SEI formation cycle.

Hankins et al. [38] combined a kinetic Monte Carlo model with DFT reaction energetics for 1 M NaPF_6_ in EC:PC and found NaF formation to be thermodynamically unfavorable under dry, pristine conditions, so that trace water or impurities are needed and the baseline SEI is dominated by Na_2_CO_3_ rather than NaF. Barnes et al. [39] confirmed this by NMR, detecting NaF and PO_x_F_y_ species that increased with water content and temperature, which shows these byproducts are soluble and evolve in the bulk electrolyte rather than staying fixed in the SEI. FEC partially suppressed PO_x_F_y_ formation, and experiments with aged electrolyte implicated the sodium counter electrode as a source of salt decomposition. In our measurements PO_x_F_y_ was absent, consistent both with this FEC suppression and with salt decomposition occurring at the counter electrode, where soluble products would not reach the working electrode in the absence of cross talk.

Depth‐resolved chemical profiles and morphological analyses suggest that SEI composition and structure can vary not only in the depth profile but also laterally across the electrode surface. Saleh et al. [31]. used scanning electrochemical cell microscopy on polished HC particles and observed SEI growth at discrete micro spots whose electrochemical properties differed from the surrounding surface, indicating local heterogeneity in SEI thickness, reactivity, or passivation. Such variation, driven by local differences in growth kinetics and substrate reactivity such as active material versus binder or conductive additive, can produce spatially varying interphases that are averaged out in spatially unresolved measurements. This integrates well with our interpretation, that rather than assuming strictly homogenous layering across the electrode, the SEI may include regions with deviating growth of different inorganic deposits as seen in EDS mappings of F in Figure 4e. This interpretation is further supported by the pronounced lateral heterogeneity observed in the topography‐corrected ToF‐SIMS analysis at the stage of full sodiation, indicating a persistent NaF‐rich surface layer with spatially non‐uniform accumulation. This may be reasoned by inhomogeneous particle surface microstructures and modifications or the formation of oxides prior to NaF formation. ToF‐SIMS depth profiling adds chemical information in our analyses, where oxygen‐rich components like carbonate and oxide/hydroxide fragments dominated the outer SEI at low potentials, whereas fluorinated species (only NaF‐fragments) increased steadily in relative intensity with sodiation and remained the major component upon desodiation. The irreversibility of NaF accumulation explains why conductivity did not recover fully upon charging, despite the apparent decrease in Na and O contents seen by EDS. This is in good agreement with DFT simulations by Alptekin et al. showing a dense inorganic layer made of NaF, which binds stronger to the HC and dominates the SEI functionality [30].

The kMC simulations by Hankins et al. [38] further reveal SEIs with very low electronic conductivity emerge as the most stable, since they hinder further parasitic reactions. The model further predicts that higher C‐rates yield thinner SEIs enriched in organic components, whereas lower rates promote thicker, more inorganic films. This agrees well with our findings of majorly inorganic SEI components after GCD at 0.1 C. c‐AFM measurements showed that electronic conductivity across the electrode surface rapidly diminishes below 0.2 V versus Na^+^/Na and is almost fully suppressed at 0.01 V versus Na^+^/Na, consistent with the formation of an electronically insulating SEI. Only sharply defined, localized conductive spots reappeared at 2.0 V versus Na^+^/Na, hinting on partial chipping or scaling as a process of delamination or cracking of the SEI. This behavior mirrors findings in Li‐ion batteries, where SEI formation effectively decouples electron and ion transport [40]. The more pronounced suppression in sodium systems has been attributed to higher inorganic content, particularly NaF [31], which have been reported to pose the favorable properties of being electrochemically inactive and electronically insulating [31, 34].

Cracking and delamination would likely accompany the volume changes associated with Na insertion and extraction in HC. These mechanical perturbations could locally rupture the SEI, exposing fresh carbon and leading to repeated electrolyte reduction, explaining the partial recovery of conductivity at 2 V versus Na^+^/Na. The *κ* values extracted from SECM approach curves provide a direct electrochemical correlation to the morphological and chemical observations. High *κ* values in the pristine state confirm facile electron transfer across the exposed carbon interface. With sodiation, *κ* decreases by several orders of magnitude, coinciding with the rise of F and O contents, the densification of the SEI in SEM, and the conductivity suppression seen in c‐AFM. During desodiation, *κ* only partially recovers, consistent with the persistence of fluorine‐rich, electronically insulating species as evidenced by ToF‐SIMS and EDS. These results support the notion that NaF is a dominant factor in limiting electron transport across the SEI, a conclusion also drawn by previous SECM and impedance studies [41, 42]. The lower reduction potential of Na^+^/Na and the less stable PF_6_^−^ in NaPF_6_ accelerate inorganic salt formation, while polymeric species typical for Li systems in SIB those are disfavored possibly associated with the weaker Lewis acidity of Na^+^ and prone to oxidation. This results in a higher fraction of Na_2_CO_3_ and NaF, producing a denser, more brittle SEI with limited elasticity, favoring delamination.

The data converge on a picture where the SEI on HC forms in two main stages: (i) rapid formation of an oxygen‐rich outer layer from solvent decomposition at potentials below 0.2 V versus Na^+^/Na, and (ii) progressive relative enrichment in NaF that yields a dense, inorganic, electronically insulating interphase. While oxide‐ and carbonate‐rich phases diminish upon desodiation, NaF persists and prevents full conductivity recovery. This inorganic‐dominated SEI contrasts with the more organic, polymeric SEIs observed in Li‐ion systems [35, 43]. The cross‐validation of structural, chemical, and functional probes in this study thus strengthens the interpretation that NaF formation is the key factor governing interfacial passivation in Na‐ion batteries. Still, partial conductivity is regained upon desodiation, for which two pathways may be considered. First, oxidic deposits could hinder NaF formation and expose fresh HC surface for NaF formation only during later cycles, owing to their higher solubility. Alternatively, if the effect were due to simple dissolution of the degradation layer, the c‐AFM maps would be expected to show a broadly increased conductivity across the measured area. Instead, conductivity reappears mainly at sharply defined spots. Taking this into account, the localized rather than area‐wide recovery would be consistent with the SEI being chipped off entirely in some regions while remaining intact elsewhere. This would point to particles with microstructural surfaces that are prone to delamination and tentatively suggests that the resistance of the NaF layer against delamination could be a key factor in forming a stable SEI. The localized nature of this recovery, and its proposed link to surface microstructure, could be verified in future work by combining imaging on identical locations with spatially resolved chemical mapping (e.g., EDS and ToF‐SIMS imaging) and AFM‐based adhesion measurements, which would directly correlate the re‐exposed conductive domains with the chemistry and mechanical stability of the underlying interphase.

The NaF‐dominated interphase observed here need not be ascribed to the fluorinated additive. NaF‐bearing interphases in NaPF_6_‐based electrolytes have been attributed to reductive decomposition of the PF_6_^−^ [44], and SEI formation on HC has been reported to be controlled largely by salt‐anion decomposition [30]. A NaF‐bearing interphase can therefore arise from the salt alone. Additive‐free electrolytes containing NaPF_6_ still develop NaF and in some cases reach comparable or higher initial Coulombic efficiency than FEC‐containing analogues [8, 12]. As the additive cannot be removed here without destabilizing the sodium counter electrode for its passivating behavior [45], the conclusions refer to the EC/PC–FEC/NaPF_6_ system studied and do not assign the observed NaF to a specific precursor.

Overall, our combined analyses indicate that the NaF‐dominated SEI, though electronically protective, remains mechanically fragile while chemically stable. If the hypothesis of delamination is confirmed, mitigating delamination and fracture of the SEI through HC and electrolyte optimization, additive selection, and formation protocols appear central to improving SIB durability. The results imply the need for HCs (1) with little to no volumetric changes during battery operation to secure the brittle NaF layer from delamination and (2) HC with surface modifications optimized to prevent NaF delamination. Furthermore, the selection of the electrolyte may fall on those including agents releasing F already at low SOC to prevent the build‐up of the oxide‐rich layer. Lastly, formation protocols are to be developed in the proximity of NaF formation potentials.

## 4 Conclusion

HC electrodes were systematically examined at defined sodiation stages to decouple anode processes from cathode influences and to capture the dynamic evolution of the SEI. By interrupting half‐cell cycling at selected cut‐off potentials, it was shown that the widespread practice of ex‐situ characterization solely in the fully desodiated state (∼2.0 V vs. Na^+^/Na) provides only a limited and potentially misleading snapshot of interfacial chemistry, which captures the SEI after it has already partially reformed or delaminated. By combining morphological (SEM), nanoscale conductivity (c‐AFM), chemical composition (XPS, EDS), depth profiling (ToF‐SIMS), and interfacial electrochemical conductivity (SECM), we can link structural, chemical, and functional changes across lateral spread and dependent on state‐of‐charge.

Across all applied analytical techniques, consistent evidence revealed that degradation products and surface layers evolve partially and reversibly and depend strongly on the electrochemical potential. SEM demonstrated the cyclic build‐up and partial removal of a surface layer, while EDS quantified the corresponding changes in Na and O abundance. ToF‐SIMS depth profiling identified NaF, Na_2_O, NaOH, and Na_2_CO_3_ as the predominant inorganic components, and c‐AFM measurements confirmed their electronically insulating character during sodiation, with conductivity recovering upon desodiation as the layer was partially lost. Critically, ToF‐SIMS further revealed that NaF is not homogeneously distributed after the first cycle, suggesting that oxidic degradation products such as Na_2_O, NaOH and Na_2_CO_3_ may passivate portions of the surface and thereby impede the formation of a uniform NaF film. To the best of our knowledge, this is the first study to directly resolve SEI composition, structure, and functionality at intermediate sodiation states during first galvanostatic cycles, to track SEI formation stepwise, and to formulate initial assumptions of delamination upon desodiation.

The results establish that SEI formation and dissolution are continuous, potential‐dependent processes rather than discrete events confined to the initial cycle. This finding refines and sharpens the current mechanistic picture in the literature: because prior studies have predominantly characterized the SEI in the fully desodiated state, they have systematically observed an interface that has already undergone partial reconstruction. For the two alternative pathways of SEI dissolution and physical delamination we propose physical delamination, rather than dissolution, as the more likely pathway, a hypothesis to be tested directly in future work. Recognizing this dynamic behavior provides critical guidance for optimizing electrode conditioning and formation protocols. By tailoring the potential window and formation rate, it may be possible to minimize irreversible electrolyte decomposition, enhance initial Coulombic efficiency, and reduce the cost and duration of industrial formation procedures for sodium‐ion batteries.

## 5 Experimental

### 5.1 Electrodes

Electrode sheets were prepared in a process adapted from LIB technology by Klemens et al. [46] and provided by collaborators P. Stüble and A. Smith form Karlsruhe Institute of Technology. In an aqueous solution HC (Kuranode Type II, 9 µm, Kuraray, Japan) was mixed with Super C65 (Imerys) and CMC (2 wt. % MAC500LC, Nippon Paper, Japan), to which in the last step styrene‐butadiene rubber dissolved in water (SBR, 40 wt. %, Zeon Europe) was added, resulting in a combined solid content of 43 wt.%. The coating and drying were also developed by Klemens et al. [47, 48] as a discontinuous quasi‐isothermal process. Using a doctor blade (ZUA 2000.60, Zehntner) the slurry was coated on an Al‐current collector, which was dried at 0.75 g m^−2^ s^−1^ using an impingement dryer and temperature‐controlled heating plates. Calendaring at 50°C of dried electrodes resulted in a final composition of the HC electrode of 93 wt. % HC, 1.4 wt. % C65, 1.87 wt. % CMC, 3.73 wt. % SBR. Electrodes were punched out of sheets as delivered and dried under vacuum at 120°C overnight in a glass oven (Model B‐585, Büchi, Switzerland). All following steps were done in an Ar filled glovebox (c(H_2_O) < 1 ppm, c(O_2_) < 1 ppm) and transported to the machines in airtight transfer vessels (VCT‐500, Leica Microsystems, Germany or transfer box, IONToF, Germany).

### 5.2 Sample Preparation

Electrochemical cells were stacked in a three‐electrode setup using Swagelok (USA) compartment. HC working electrodes were punched out from the delivered sheets with 12 mm diameter to reach 4 mg active material with 1 C capacity. Counter and reference electrodes were rolled and punched out (12 mm, counter electrode) or smeared (reference electrode) from Na‐metal (99.9%, BASF, Germany), respectively. 70 µl of 1 M NaPF_6_ in a mixture of EC:PC (1:1) to which 5 wt.% FEC were added (E‐Lyte, Germany) was used as electrolyte. Separators were punched out from glass fibre sheets (Type A, Whatman, UK) of which one of each 12 and 13 mm were used per cell to strictly prevent contact between working‐ and counter electrode. Electrochemical GCPL (12 h OCV followed by 60 µA charging and discharging to the respective cut‐off potentials) and CV (50 mV/s, 5 cycles) was carried out using a VMP300 potentiostat (BioLogic, France) together with the software BT‐Lab (V1.81).

After disassembly, electrodes were rinsed (3×0.5 mL DMC, dried over mole sieves, 4 µm, < 10 ppm H_2_O) and dried under vacuum overnight in a glass oven (B‐585, Büchi, Switzerland) at room temperature. A transfer system from Leica company (VCT 500) was used for sample transfer between the glove box and the analytical devices to maintain the inert atmosphere and to avoid surface reactions.

### 5.3 SEM/EDS

Scanning electron microscopy and energy dispersive spectroscopy: Electron images were obtained using a high‐resolution field‐emission SEM instrument Gemini SEM 560 (Carl Zeiss Microscopy GmbH, Germany) equipped with an *Extreme* x‐ray detector (Oxford Instruments, United Kingdom) operated by Aztec 6.1 software package (Oxford Instruments, United Kingdom). Electron images were recorded at 5 mm working distance, 2 kV excitation voltage and a beam current of 5 µA. Image processing was done using ImageJ (Version 1.54p). EDS quantification of light elements (O, F, Na) was performed at 3.5 kV. At this accelerating voltage, all elements of interest are adequately excited, with overvoltage ratios *U* = *E*_0_/*E*_c_ ranging from 3.4 (Na Kα) to 12.3 (C Kα), ensuring reliable characteristic x‐ray generation. All samples were transferred air‐free prior to analysis to prevent surface oxidation artifacts. As standardless EDS quantification of light elements carries inherent uncertainties, results are interpreted as semi‐quantitative, with emphasis placed on relative trends across SOC rather than absolute compositions. The consistency of F trends with XPS data (cf. Figure S5) supports the validity of the observed SOC‐dependent evolution in surface chemistry. Mappings were done with a resolution of 1024 × 1024 pixels, a dwell time of 100 µs and 5 frames per measurement.

### 5.4 (AFM‐) TOF‐SIMS

Samples were introduced to a ToF‐SIMS M6 (IONToF, Münster, Germany) on a backmount sample holder. SIMS depth profiles in spectrometry mode were obtained using Bi_3_^+^ (*I* = 0.06–0.08 pA) as primary ion species on an analysis area of 50 × 50 µm^2^ at 128 × 128 pixels centred to a sputter area of 300 × 300 µm^2^. For sputtering a 10 keV Ar_x_^+^ beam with a cluster size distribution at Ar_1000_^+^‐ions (cf. Figure S8) and a beam current of 8.00 nA was used. A cycle time of 100 µs was chosen for the non‐interlaced mode of sputtering and analysis, during which each analysis frame was followed by one sputter frame and 0.5 s of pause time for charge relaxation. Stop condition was a limit of 300 s sputtering at which a plateau region resembling the HC particle was reached. To ensure reproducibility, all TOF‐SIMS measurements were carried out at least twice on independently prepared samples. One dataset is presented throughout this work, while raw data for both sets is available, c.f. Data Availability Statement.

An additional HC sample sodiated to 0.01 V was introduced into the machine on a top‐mount sample holder for correlative AFM‐SIMS analysis for which the primary ion gun was operated in fast imaging mode using 20 keV Bi_3_^+^ clusters (*I* = 0.019 pA at a cycle time of 100 µs and a beam defining aperture of 110 µm). The ToF‐analyser was operated in imaging mode with nominal mass resolution, and no electron flooding was applied to avoid introducing measurement artifacts to the electronically conductive sample. The analysis beam was scanned in random mode, and 256 × 256 pixels were measured on an area of 35 × 35 µm^2^. The dose density was kept below 10^12^ ions·cm^−2^ per scan to maintain static conditions. Sputtering was carried out as described above on an area of 135 × 135 µm^2^. A total of 70 scans were obtained with each scan consisting of 10 analysis frames, 1 sputter frame and 0.5 s charge relaxation. Before and after the acquisition, the topography of the sample surface (30 × 30 µm^2^, 2048 × 128 pixels) was scanned in intermittent contact mode with the SPM (Figure S9). The depth was evaluated with a line scan of a second crater, which was produced with the same sputter settings (Figure S10).

Data evaluation was carried out using Surface Lab 7.5 (IONToF, Münster, Germany), fragments of Na_2_^+^, Na_2_OH^+^, Na_2_F^+^ and Na_3_CO_3_^+^ were used for mass calibration in positive ion mode.

### 5.5 C‐AFM

Conductive Atomic Force Microscopy (c‐AFM) measurements were carried out to investigate the local electronic conductivity of the electrode surfaces using a Park NX10 AFM (Park Systems, South Korea) housed within an Ar‐filled glovebox (Unilab, MBraun, O_2_< 0.1 ppm, H_2_O < 0.1 ppm). Single‐crystal conductive diamond probes (AD‐2.8‐AS, single‐crystal diamond probes (AD‐2.8‐AS, Oxford Instruments, UK) with a nominal spring constant of 2.8 N/m and a resonant frequency of 65 kHz were employed to provide high durability and high wear resistance. Measurements were performed in contact mode over areas of 30 × 30 µm^2^, recorded at a resolution of 512 × 512 pixels. The samples were mapped with a scan velocity of 0.3 Hz, and a bias of 2.5 V was applied between sample and AFM probe. For detailed analysis, selected sub‐regions of 12 × 12 µm^2^ were extracted at 205 × 205 pixels resolution, enabling localized assessment of conductivity variations across different surface features.

### 5.6 SECM

Approach curves of pristine and cycled HC composite electrodes were acquired in feedback mode using an electrolyte containing 1 M NaPF_6_ and 20 mM Fc in EC/PC. A Pt UME (*r* = 1.5 µm) was used as the working electrode, an AgCl‐coated silver wire served as quasi‐reference electrode, and a Pt wire functioned as the counter electrode. All measurements were performed with a Sensolytics SECM system (version 3.12, Sensolytics, Germany). The applied tip potential was 0.4 V versus AgQRE. Approach curves were recorded at an approach speed of 2 µm s^−1^ and analyzed using the SECM finite sample kinetics model [32] in MiRa (Version 2.0, G. Wittstock, University of Oldenburg, Germany). Figure S7 presents the fitted approach curves for each sample condition, with the associated *κ* values obtained from the fits.

### 5.7 XPS

The x‐ray photoelectron spectroscopy measurements were conducted with a Versaprobe IV (ULVAC‐PHI, Inc., Chanhassen, USA). A monochromatized Al K_α_ x‐ray source was employed (beam diameter of 200 µm, x‐ray power of 50 W, 15 kV) together with charge neutralization by slow electrons while the pressure in the XPS chamber was in the range of 10^−7^ to 10^−6^ Pa. A step time of 50 ms, a step size of 0.200 eV, and an analyser pass energy of 27 eV were used for the detailed spectra. Transport in a transfer shuttle minimized cross contaminations with atmospheric moieties. Data analysis was carried out with the *CasaXPS* software (Version 2.3.26, Casa Software, Devon, UK), where charge correction was done using C 1s (284.8 eV) if possible and F 1s (684.27) otherwise and a Shirley background was subtracted from the raw data.

## Conflicts of Interest

The authors declare no conflict of interest.

## Supporting information




**Supporting File**: smtd70958‐sup‐0001‐SuppMat.docx.

## Data Availability

Data Availability Statement The data that support the findings of this study are openly available at Zenodo at https://doi.org/10.5281/zenodo.20053613.
