# Supplementary material for: The First Electrochemical Cycle: State‐of‐Charge Dependent Formation and Evolution of the Solid Electrolyte Interphase on Hard Carbon Anodes in Sodium‐Ion Batteries
Source: Small Methods. 2026 Aug 14;10(18):e70958. doi: 10.1002/smtd.70958 (PMC13599700; doi:10.1002/smtd.70958)
Supplement: Supplementary file 1 [file SMTD-10-e70958-s001.docx]

## Supporting Information

# *The First Electrochemical Cycle: State-of-Charge Dependent Formation and Evolution of the Solid Electrolyte Interphase on Hard Carbon Anodes in Sodium-Ion Batteries*

*David Schäfer^a^, Sven Daboss^b^, Sarah Lang^a^, Christine Kranz^b^, Marcus Rohnke^a^**

^a^ Institute of Physical Chemistry and Center for Materials Research, Justus Liebig University Giessen, Heinrich-Buff-Ring 17, 35392 Giessen, Germany.

^b^ Institute of Analytical and Bioanalytical Chemistry, Ulm University, Albert-Einstein-Allee 11, 89081 Ulm, Germany.

Figure S1: GCD measurements (half cell potential E_WE_ versus capacity C) of investigated sodiation stages a) during the first cycle and b) after 4 cycles of HC electrodes cycled against sodium in a three electrode setup at 0.1 C (65 µA, 58 µA/cm^2^). Comparability of the different cells is given by the similarity of the GCD curves, especially during the first cycle.

Figure S2: SEM images of HC surfaces at different cut-off potentials. NPAC stands for “non-polarized assembled cell” in the pristine state. 1.2 V to 0.01 V refer to the sodiation branch, while 0.01 V to 2.0 V refer to the desodiation branch. The surface of the particles gets inreasingly covered with sodiation, which is partly reverted upon desodiation.

Figure S3: c-AFM measurements of HC surfaces at different cut-off potentials. NPAC stands for “non-polarized assembled cell” in the pristine state. 1.2 V to 0.01 V refer to the sodiation branch, while 0.01 V to 2.0 V refer to the desodiation branch. With incresing sodiation the surface of the particles becomes less conductive. During desodiation the conductivity is partly recovered.

Figure S4: c-AFM topography maps of HC surfaces at different cut-off potentials. NPAC stands for “non-polarized assembled cell” in the pristine state. 1.2 V to 0.01 V refer to the sodiation branch, while 0.01 V to 2.0 V refer to the desodiation branch. The topography images correspond to the current maps presented in Figure 3f-j and Figure S3.

Figure S5: High-resolution F1s XP spectra of HC electrodes sodiated to different potentials (NPAC, 0.2 V, 0.01 V, 0.3 V, and 2.0 V vs. Na^+^/Na). Spectral deconvolution reveals the relative contributions of NaF (~ 684.5-685.0 eV) and residual NaPF_6_ salt (~ 687-688 eV). The NPAC sample shows a mixed surface chemistry with NaPF_6_ remnants and NaF possibly originating as cross talk degradation product from the Na metal coutner electrode. Sodiated HC electrodes exhibit a dominant NaF signal, consistent with extensive salt decomposition and the formation of an inorganic, fluoride-rich SEI.

Figure S6: Cumulative ion spectrum of the whole depth profile in positive ion mode of an electrode sodiated to 0.01 V vs. Na^+^/Na normalized to total primary ion dose. Major peaks are marked with the respective ion fragments: Na_2_O, NaOH, NaF and Na_2_CO_3_ as the major SEI forming degradation products. The ion C_2_Na_3_^+^ can be seen as a fragment from the sodiated carbon, which is backed by its occurrence at higher sputter fluences in Figure 4h (0.01V).

Figure S7: a) SECM approach curves recorded in the presence of ferrocene as redox mediator at HC electrodes at different cut-off potentials (NPAC, 0.2 V, 0.01 V, 0.3 V, and 2.0 V vs. Na^+^/Na). The variation in approach behavior reflects changes in interfacial electron-transfer kinetics and surface reactivity as a function of the electrochemical state of the electrode. The NPAC sample exhibits positive feedback, indicative of a well conductive electrode surface. In contrast, electrodes sodiated to defined potentials show a significantly suppressed current signal, consistent with the formation of an electrically passivating SEI. E_tip_ = 0.4 V vs. AgQRE in 1 M NaPF_6_ / 20 mM ferrocene in EC/PC, v = 2 µm s^−1^ b) Schematic representation of the SECM measurement principle, illustrating the approach of the ultramicroelectrode (UME) toward the sample surface (L = d / r_tip_). At large tip-sample separations, the redox mediator can freely diffuse to the UME, resulting in a diffusion-controlled current characteristic of semi-infinite conditions. As the tip is brought into close proximity to the surface, diffusion becomes increasingly hindered, and the measured current becomes sensitive to the ability of the sample to regenerate (i.e., reduce) the electroactive species.

Figure S8: Ar_n_^+^ cluster-size distribution used during ToF-SIMS depth profiling. Measured current values for Ar_n_^+^ clusters are shown as a function of cluster size. The distribution exhibits a broad maximum around n ≈ 1000, consistent with the typical emission characteristics of the GCIB source under sputter operating conditions.

Figure S9: SPM topography images acquired on the same electrode area (a) before and (b) after ToF-SIMS depth profiling. The pre-profiling image shows the native surface morphology prior to ion sputtering, showing granular features on the micrometer scale. After sputtering these structures have seemingly been smoothed out.

Figure S10: a) AFM line-scan analysis acquired before (blue) and after (red) SIMS depth profiling and b) point-by-point height difference, highlighting the net material removal induced by ion sputtering. The shaded region marks the interval used for quantitative crater-depth evaluation, with respect to the reference interval at x = 250 µm ± 10 µm. Vertical markers (red) indicate the boundaries of the SIMS crater which was calculated to have an avergage depth of 788 nm.
